# Functional Neurological Disorder: A Case of Twisted Toes and Dystonia

**DOI:** 10.7759/cureus.88648

**Published:** 2025-07-24

**Authors:** Regina J McPherson, Juan R Santos-Rivera, Ilya Fonarov

**Affiliations:** 1 Medicine, Florida International University, Herbert Wertheim College of Medicine, Miami, USA; 2 Internal Medicine, Ponce Health Sciences University, Ponce, PRI; 3 Internal Medicine, Jackson Memorial Hospital, Miami, USA

**Keywords:** dissociative (conversion) disorder, dystonia, factitious disorder imposed on another, functional neurologic disorder, malingering, psychogenic

## Abstract

Functional neurological disorders (FNDs) continue to pose diagnostic challenges, often resembling organic neurological conditions and leading to extensive but unnecessary testing. FNDs commonly present with motor and sensory symptoms that fluctuate and improve with distraction, favoring differentiation from structural diseases. This case describes a 53-year-old female with bilateral lower extremity pain and toe contractures, initially concerning for an organic movement disorder or peripheral neuropathy. Examination revealed distractible dystonia, normal strength, and negative neuroimaging, supporting a diagnosis of FND. Despite prior similar presentations, diagnostic uncertainty and the presence of comorbid conditions such as diabetic peripheral neuropathy complicated the evaluation. Our report emphasizes the importance of recognizing positive clinical signs, reducing reliance on exclusion-based diagnosis, and addressing stigma to improve patient care and management.

## Introduction

Functional neurological disorders (FNDs) present a diagnostic challenge as they can mimic acute neurological deficits requiring urgent evaluation. FMDs, a motor subtype of FND, are a key clinical manifestation. These symptoms are truly experienced by the patient and typically present as one or more motor deficits that are inconsistent with recognized neurological diseases and lack a known anatomical pattern [1]. It is important to distinguish FNDs from malingering and factitious disorders, which involve deliberate deception. FND was labeled as a conversion disorder, psychogenic disorder, or dissociative disorder [2].

FNDs result from dysfunction in brain networks rather than structural abnormalities, and neuroimaging studies often appear normal, making the diagnosis even more challenging [1,3]. Functional dystonia is one of the core phenotypes of FMD and is frequently characterized by fixed postures, inconsistent resistance, variability with attention, and abrupt onset [2,4]. According to Galli et al., common bedside clues for functional dystonia include sudden onset, fixed abnormal postures, and improvement with distraction [3].

Epidemiologically, dystonia is the second most common subtype of FMD after tremors [2,5]. Patients with functional dystonia often have comorbidities, such as chronic pain, fatigue, or anxiety [5]. The demographic profile typically includes a female predominance and presentation in early to mid-adulthood [6]. In children and adolescents, dysfunctional family dynamics or psychological stressors can be relevant, but abuse is less frequently implicated [2,7].

Functional dystonia must be distinguished from organic dystonia, which is usually gradual in onset, progressive, and often painful. Functional dystonia is typically non-progressive and more likely to show variability, entrainment, or inconsistency on physical examination [4,8]. According to the Diagnostic and Statistical Manual of Mental Disorders, Fifth Edition, Text Revision (DSM-5-TR), FND is classified under “somatic symptom and related disorders,” and diagnosis is based on positive clinical signs that are incompatible with recognized neurological conditions [9].

Patients with functional dystonia often face stigma, including misdiagnosis or minimization of symptoms. This can worsen outcomes by delaying appropriate treatment [10].

## Case presentation

A 53-year-old woman, with a past medical history of hypertension, long-standing type 2 diabetes mellitus complicated by peripheral neuropathy, FND, cannabis, benzodiazepine, amphetamine, phencyclidine and opioid use disorder, chronic kidney disease, asthma/chronic obstructive pulmonary disease (COPD), and anxiety disorder, presented to the emergency room (ER) for pain in her lower extremities and bilateral contractures in her toes. She noticed a gradual onset over a week where she experienced bilateral lower extremity throbbing pain, which did not improve with acetaminophen. She then noticed her toes were flexed, resulting in an inability to ambulate. She denied any other neurological complaints. The patient had a recent admission to the hospital three months prior for facial droop, dysarthria, progressive bilateral extremity weakness, and numbness associated with bilateral flexion contraction of the toes. An extensive workup, including CT and MRI brain, was negative for acute stroke; imaging of the neuroaxis was negative for structural abnormalities or demyelinating lesions. Given her psychiatric history, negative findings on work-up, and improvement in her symptoms with distraction on examination, it was suspected that her presentation was due to functional neurological disorder. Her symptoms at that time further improved with home physical therapy.

**Figure 1:**
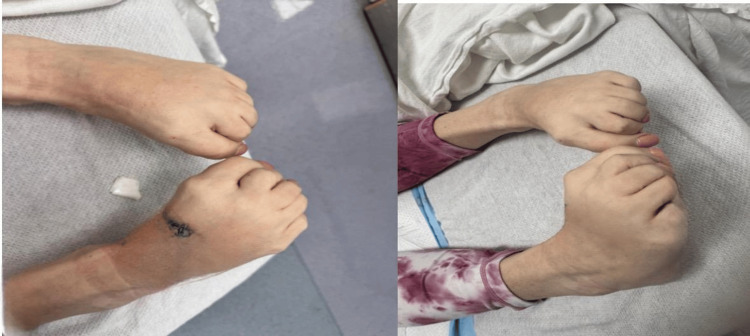
Inversion and plantar-flexion of the bilateral feet and toes.

On evaluation, her vital signs were within normal limits. Her exam was notable for 5/5 strength bilateral upper and lower extremities, negative pronator drift, and intact light touch, but decreased vibration sense in bilateral lower extremities. Reflexes were normal, notably with a flexor plantar reflex. Her review of systems was unremarkable. Inversion and plantar flexion of the bilateral feet were noted (Figure 1). The patient was unable to ambulate due to flexion of her feet and toes. It was notable that the patient’s dystonia altered with distraction; her toe plantar flexion improved. All laboratory work-up was within normal limits. No new imaging was ordered; however, her MRI brain one month before was without any acute abnormalities. Neurology was consulted for recommendations and concluded that the patient’s flexion was distractible and consistent with her prior diagnosis of FND.

A discussion of FNDs was completed by the neurology team with the patient, explaining what it meant, the possible variation in presentation, risk factors, and treatment. The patient was discharged with home physical therapy and outpatient neurology follow-up.

## Discussion

The key distinguishing feature of FNDs is the presence of neurological symptoms that are inconsistent with structural or pathophysiological disease processes [1,2]. FNDs can mimic a wide spectrum of organic neurological conditions, including stroke, epilepsy, and movement disorders [3,5].

Diagnosis relies not on exclusion alone, but on the identification of positive clinical signs and a detailed history that uncovers inconsistencies and functional features [2,4]. Eliciting symptom onset, progression, and potential triggers can highlight associations with psychological stressors or trauma [8,9]. FND is frequently linked to psychiatric comorbidities, such as anxiety, depression, or a history of adverse life events, making psychiatric and psychosocial history an important part of the assessment [1,7]. Bedside signs such as Hoover’s sign, collapsing weakness, co-contraction, and entrainment of tremor support the diagnosis of motor FND [4]. Gait disturbances and psychogenic nonepileptic seizures (PNES) also exhibit distractibility and inconsistency in symptom patterns [6]. According to the Diagnostic and Statistical Manual of Mental Disorders, Fifth Edition, Text Revision (DSM-5-TR), FND is classified under somatic symptoms and related disorders and requires at least one motor or sensory symptom that is incompatible with recognized neurological conditions [9]. While diagnostic imaging and labs are typically normal, EEG, MRI, or EMG may be used selectively to rule out organic pathology [2,10].

In our patient, the presence of recurrent neurological symptoms, normal imaging, and inconsistencies on the examination strongly supported a diagnosis of functional dystonia. Her prior admission for similar symptoms further reinforced the diagnosis. Despite a reported inability to ambulate, the absence of corresponding deficits on examination highlighted the functional nature of her presentation. Neurology confirmed the diagnosis without additional testing, helping avoid unnecessary investigations and enabling the clinical team to focus on education, physical therapy, and outpatient support.

FND encompasses a broad spectrum of motor and sensory symptoms that fluctuate and lack anatomical consistency [2,4]. Tremors, gait disturbances, and limb weakness are most common, while focal dystonia and sudden paralysis are less frequently reported [3]. Luba et al. described a case of psychogenic lower limb paralysis following bereavement, with extensive negative testing preceding the diagnosis [11]. Nehete et al. reported isolated head titubation in a postoperative setting that resolved with cognitive-behavioral therapy (CBT) [12], while Kyriazidis et al. documented acute functional hand paralysis after minor trauma with rapid spontaneous recovery [13].

In contrast to the previously described cases, our patient presented with a rare manifestation: isolated, recurrent toe dystonia with persistent inability to ambulate despite normal imaging, labs, and the presence of clear positive functional signs. Her prior FND diagnosis facilitated earlier recognition and timely redirection toward outpatient rehabilitation. This case underscores the importance of identifying positive clinical signs such as distractibility and inconsistency to avoid unnecessary testing.

A crucial first step is delivering a clear, supportive explanation of the diagnosis, which has been shown to improve patient outcomes [3]. For motor symptoms such as dystonia, targeted physical therapy helps retrain movement patterns, while CBT addresses psychological comorbidities and supports long term recovery [5,10]. In this case, early diagnostic clarity allowed for prompt referrals, likely reducing the risk of chronic disability and healthcare overutilization.

To assist clinicians in distinguishing FND subtypes from their organic counterparts, Table 1 outlines key clinical features and bedside findings, including those demonstrated in our patient’s presentation of functional dystonia.

**Table 1:** Key distinguishing features of functional neurologic disorders and similar organic conditions.

Functional Neurologic Disorder	Similar Organic Conditions	Key Differentiating Features	References
Functional Weakness	Stroke, multiple sclerosis (MS), myopathy	Hoover’s sign (weakness improves with contralateral activation), variability in strength, non-anatomical distribution	Peeling et al., 2023 [1]
Functional Tremor	Parkinson’s disease, essential tremor	Tremor changes with distraction, entrainment present, variability in amplitude/frequency	Bartl et al., 2020 [14]
Functional Dystonia	Dystonia (genetic or acquired), tardive dystonia	Sudden onset, inconsistent posturing, improvement with distraction	Frucht et al., 2021 [10]
Functional Gait Disorder	Cerebellar ataxia, Parkinsonian gait, peripheral neuropathy	Exaggerated, bizarre movements (astasia-abasia), improvement with distraction, lack of true falls	Perez et al., 2021 [15]
Functional Sensory Loss	Peripheral neuropathy, spinal cord lesions	Non-dermatomal distribution, splitting midline, variability on repeated testing	Anderson et al., 2019 [16]
Functional Vision Loss	Optic neuritis, retinal disease, hysterical blindness	Normal pupil responses, preserved optokinetic reflex, inconsistent confrontation testing	Manion et al., 2025 [17]
Psychogenic Non-epileptic Seizures (PNES)	Epilepsy (focal or generalized)	Eyes closed, asynchronous limb movements, prolonged duration, no post-ictal confusion, normal EEG	Huff et al., 2025 [18]
Functional Myoclonus	Cortical myoclonus, spinal myoclonus	Variable or stimulus-sensitive jerks, inconsistent pattern, distractibility	Van der Veen et al., 2022 [19]
Functional Syncope	Cardiac syncope, orthostatic hypotension	Atypical triggers, prolonged unresponsiveness without injury, normal cardiac workup	Huff et al., 2025 [18]
Functional Cognitive Disorder	Dementia, mild cognitive impairment	Subjective complaints but normal objective testing, inconsistencies in cognitive deficits	Peeling et al., 2023 [1]
Functional Dysphonia	Vocal cord dysfunction, neurologic dysphonia	Sudden onset, improvement with distraction, no structural vocal cord abnormalities	Peeling et al., 2023 [1]
Functional Stuttering	Neurogenic or developmental stuttering	Symptoms worsen with attention but improve with distraction	Peeling et al., 2023 [1]

## Conclusions

This case illustrates the diagnostic challenge of FND, particularly when symptoms such as focal dystonia mimic organic pathology. The patient’s distractible toe posturing, preserved strength, and unremarkable imaging supported a positive diagnosis of FND, allowing the care team to avoid unnecessary testing and shift promptly toward symptom-directed management. Coexisting conditions such as diabetic neuropathy complicated the evaluation, reinforcing the need for a thorough history and focused neurological examination. Functional and organic disorders can coexist, and recognizing both components is essential for accurate diagnosis and comprehensive care. Recognition of functional signs at the bedside remains essential for timely diagnosis and appropriate referral to physical therapy, psychiatry, or neurology as needed. While stigma can still contribute to delays in care, clinician familiarity with FND improves diagnostic accuracy and supports early, effective intervention.
